# *Mul*Ti-FAST: A Machinability Assessment of Functionally Graded Titanium Billets Produced from Multiple Alloy Powders

**DOI:** 10.3390/ma15093237

**Published:** 2022-04-30

**Authors:** Oliver Levano Blanch, Daniel Suárez Fernández, Alex Graves, Martin Jackson

**Affiliations:** 1 Department of Materials Science and Engineering, The University of Sheffield, Sir Robert Hadfield Building, Mappin St., Sheffield S1 3JD, UK; danillaneru@gmail.com (D.S.F.); agraves1@sheffield.ac.uk (A.G.); martin.jackson@sheffield.ac.uk (M.J.); 2 Advanced Manufacturing Research Centre, Advanced Manufacturing Park, Catcliffe, Rotherham S60 5TZ, UK

**Keywords:** machinability, diffusion bonding, field assisted sintering technology, titanium, cutting forces, turning

## Abstract

Technological developments in the area of functionally graded multi-material manufacture are poised to disrupt the aerospace industry, providing the means for step-change improvements in performance through tailored component design. However, the challenges faced during the downstream processing, i.e., machining of such functionally graded multi-materials are unclear. In this study, the challenges involved when face-turning billets consisting of multiple alloys are assessed. To achieve this, a cylindrical billet consisting of Ti-64, Ti-6242, Ti-5553 and Beta C alloys was manufactured from powder feedstock using field-assisted sintering technique (FAST) and termed *Mul*Ti-FAST billets. A detailed study of the structural integrity during machining at the diffusion bond interfaces of multiple titanium alloy bond pairings in the *Mul*Ti-FAST billet was conducted. The machining forces were measured during face-turning to investigate the impact and behaviour of different alloy pairings during a continuous machining operation. The results showed the significant differences in force machining response, surface topography and the type of surface damage was dependent on the direction the titanium alloy graded pairings were machined in. In terms of subsurface microstructural damage, regardless of the machining direction, no critical damage was found in the vicinity of the bonded alloys. The findings provide an insight into the deformation characteristics and challenges faced in the machining of functionally graded components with multiple titanium alloys.

## 1. Introduction

### 1.1. Functionally Graded Materials (FGM) and Multi-Material Components

Engineering design is often restricted by the fact that individual alloys present a narrow range of material properties. In most cases, it would be advantageous to design complex components from materials which have specific properties at different subcomponent locations. The concept of multi-material component designs and the manufacturing of such materials presents an interesting challenge: locally defining the best material selection based on the specific loading, contact and thermal conditions calculated for the designed part [1]. Pursuing this new strategy opens the possibility of a more efficient use of resources and optimisation of the final component’s performance compared to the more traditional, single material, manufacturing methods. In the case of metallic materials, the manufacturing of newly engineered parts presents its own challenges, such as the following: mechanical and chemical compatibility; the joining method to achieve a significant improvement in the properties of interest; how to physically locate the different alloys at the required subcomponent locations; the electro-chemical compatibility of the alloys selected; the influence on downstream processes such as forging or machining; even the recyclability of such proposed multi-material and FGM components.

### 1.2. Examples of Current Manufacturing Methods for FGM

The manufacture of multi-material components has been achieved at different levels of complexity using a range of advanced metals processes. The most common technology utilized for joining dissimilar metals is welding, in particular, variants of metal arc welding [2]. During welding, the high energy needed to join the two alloys can create metallurgical incompatibility at the joint region, as reported by Sun and Karppi [3]. This can compromise the mechanical properties at the weld region, presenting heterogeneities, such as lower ductility at the interface due to the thermal cycle applied. Moreover, these techniques have a limited capability when it comes to geometrically engineering an alloy in a specific subcomponent region.

For the cases where inertia friction welding (IFW) is used, friction is used to heat the interface and create a solid-state bond between two alloys. Compared to fusion welding, IFW creates a smaller heat affected zone (HAZ) [4] and produces a better joint quality [5]. Recent developments by Rajan et al. [6] successfully demonstrated the joining of the dissimilar titanium alloys Ti-64 (Ti-6Al-4V) and Ti-6242 (Ti-6Al-2Sn-4Zr-2Mo-0.1Si), with a combined multi-material thermal affected zone (TMAZ) of ~1mm. However, this welding technology also presents similar geometric limitations. In addition, IFW creates high residual stresses at the joint that can have a deleterious effect on the mechanical properties [7] without an appropriate heat treatment. 

Wire-arc additive manufacturing (WAAM) is a hybrid process that uses an electric arc to heat up the material [8], building components layer by layer, significantly reducing the geometric limitation compared to welding. However, WAAM components tend to present poorer mechanical properties with respect to standard manufacturing methods and the cost of the wire feedstock is prohibitive (>GBP 250 per kg) [9]. The twin WAAM (T-WAAM) process takes this one step further using two alloy feed wires during manufacturing. Yang et al. [10] demonstrated that the T-WAAM arrangement with commercially pure (CP) titanium and aluminium as the feed wires, in combination with a niobium film, can be used to create a tailored alloy of composition Ti-6Al-7Nb through this processing route. In addition, Adinarayanappa and Simhambhatla [11] showed that local hardness levels can be tailored at different locations by using two different composition steel filler wires. Wang et al. [12] demonstrated the successful manufacturing of a component with a compositional gradient from pure titanium to an alloy composed of Ti-50 at.% Al, using such technology. There has also been research regarding the machinability of WAAM components made from titanium [13] and steel [14,15]. However, none of these studies have investigated the machinability of multi material components. 

Additive manufacturing (AM) provides versatility during the production of multi-material workpieces, whereby the precise positioning of powders in specific locations enables parts with complex geometries to be created from multiple alloys. For example, Zhai et al. [16] demonstrated that 3D printing can be used to manufacture a bimetal part consisting of Ti–48Al–2Cr–2Nb and Ti-6Al-4V. In their work, they found the bond integrity of the bimetal samples to be poor, as they fractured at the interface layer during a standard tensile test. This indicates that production of multi-material components using AM still requires further development before it can be scaled-up and commercialised. Moreover, all multi-material 3D printed components still exhibit poorer mechanical properties with respect to more conventional manufacturing processes due to defects such porosity, residual stress and microstructural heterogeneities [17]. 

Spark plasma sintering (SPS) or field-assisted sintering technology (FAST), is a promising technique that could enable multi-material manufacturing. FAST uses pulsed electrical current and mechanical pressure to fully consolidate powder feedstock into a fully consolidated shaped part. Dissimilar powders can be distributed in different locations of the graphite dies to obtain a multi-material component [18,19]. In comparison with conventional solid-state technologies, FAST is capable of fully consolidating the powder with short or no dwell times and lower processing temperatures [20,21]. It can also process a wide range of titanium alloy feedstocks from surplus and oversized AM powder to machined swarf [22]. Furthermore, previous studies have shown that it is possible to manufacture titanium shaped parts using FAST [23,24,25,26], as well as combining it with a single stage hot forging process (and termed as FAST-*forge* [27,28,29,30]) to obtain a near-net shaped component in only two solid-state processing steps. As such technology is being translated and scaled-up into industry, it could lead to an important reduction in the cost of titanium parts. There has been some research on using FAST to join two solid titanium blocks [31] and joining titanium with other materials with very good results [32,33,34]. In addition to this, FAST has been exploited to join dissimilar titanium powder alloys into billets and parts with excellent controlled microstructures and mechanical integrity [18,19].

### 1.3. Post-Processing Manufacturing Challenges of Multi-Material Components

This research aims to evaluate and understand the potential manufacturing challenges that arise from the manufacturing of multi-material components during important downstream processes, such as machining. To investigate the machinability characteristics of such materials, cutting forces were measured, as they have been shown to be effective when investigating the machining of bimetallic components manufactured via inertia friction welding [35,36]. In these studies, the sample pairings comprised the following: aluminium alloy A1070 and CP titanium; aluminium alloy A5052 and ductile cast iron; stainless steel SUS304 and mild steel S15CK. Ullah et al. [36] observed better results when machining from a soft to a hard material; however, the study lacked any microstructural analysis to help determine the mechanistic nature of such an effect. Furthermore, Ullah et al. [37] also showed that the surface level after traversing the bond decreased when machining from aluminium to titanium but not when machining in the other direction. 

Other studies [38,39,40,41] have investigated the machining of Al-cast iron multi-material pistons, highlighting the difference in the distinctive force response between the alloys and the accelerated tool wear rates when machining multi-material components. Moreover, Malakizadi et al. [42] concluded that the thermal cracking phenomenon was the main cubic boron nitride (CBN) tool wear mechanism during milling of bimetallic engine blocks. However, it is important to note that this work did not assess the subsurface damage or the actual surface finish of the sample. The damage occurring at the bond can have an important influence on the mechanical properties of the component, especially when they are subject to a dynamic loading regime. This is deducted from studies in monolithic alloy components [43]. Therefore, as this is a relatively unexplored research topic, the intention of this study is to provide a mechanistic understanding of the response of multi-materials during the important finish machining stage.

In this study, the analysis is focused on different titanium alloys where subtle differences in chemistry and yield strength exist, rather than distinctly different metallic systems. In order to define smart machining strategies for multiple, dissimilar titanium alloy materials, the machining dynamics and subsurface damage at the bond region with respect to machining direction were studied in detail. Machining directionality is analysed to decouple the physical and dynamic effects in the machining forces as a function of the machining direction with respect to the alloy type. The use of FAST as a processing technology to manufacture the multiple titanium alloy billets (termed *Mul*Ti-FAST) was selected because the solid-state FAST process creates homogeneous and chemically graded bonds with a minimal diffusion bonded region (<300 µm) [18,19]. Therefore, the data and conclusions derived from this study can be directly attributed to the compatibility of the titanium alloy combinations. In other techniques, where comparable commercial mechanical properties are achieved, such as welding, relatively large transition regions with a HAZ and fusion affected zone are created. This means that the bond region can be considered and analysed as a third material with distinct properties from the parent dissimilar materials.

## 2. Materials and Methods

**Figure 1:**
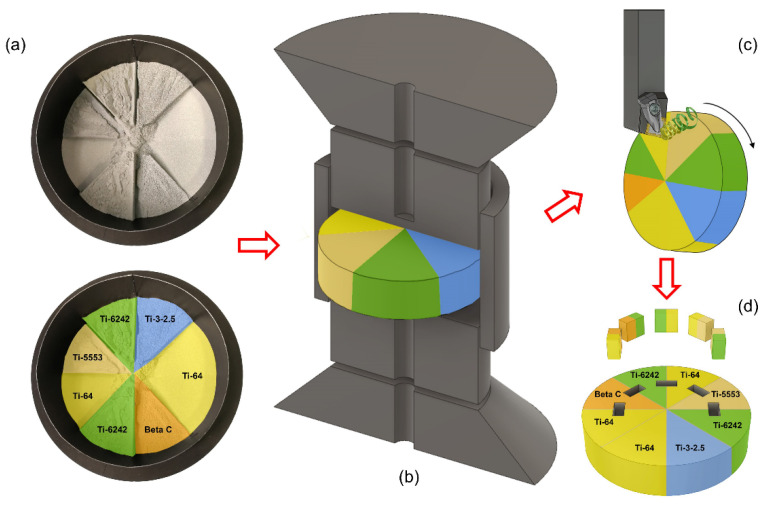
Schematic diagram of the methodology used in this research to understand the machinability of multi material components. (**a**) A photograph of five different powders distributed inside a graphite mould. (**b**) A 3D render of the cross section of the tooling used to process the powder into a solid *Mul*Ti-FAST billet. (**c**) A 3D render of the machining stage performed on the *Mul*Ti-FAST billets. (**d**) Graphic representation of the location where the material was sectioned and analysed.

The workflow followed in this work to study the machinability of multi-materials components is summarized in Figure 1. Figure 1a shows the initial stage where the dissimilar powders are poured into a graphite mould. Then, the tooling with the powder is processed using field-assisted sintering technology (FAST), as shown in Figure 1b. After making the *Mul*Ti-FAST billets, these are machined in a turning operation and subsequently sectioned for further analysis, as shown in Figure 1c,d. This section will explain in more detail the steps described in Figure 1.

### 2.1. Sample Manufacturing

The functionally graded titanium alloy *Mul*T*i*-FAST billet was manufactured for this study using FAST. The cylindrical graphite mould used was 80 mm in diameter and created solid titanium billet discs of approximately 20 mm in height.

In this study, the four titanium alloys used were: Ti-6Al-4V (Ti-64), Ti-6Al-2Sn-4Zr-2Mo (Ti-6242), Ti-5Al-5V-5Mo-3Cr (Ti-5553) and Ti-3Al-8V-6Cr-4Mo-4Zr (Beta C). The selected alloys belong to a different type of titanium alloy. Ti-6242 is a near-α alloy commonly used in the aerospace sector, Ti-64 is an α + β alloy and the most common titanium alloy, Ti-5553 is a near β alloy also used in the aerospace sector and Beta C is a β alloy with a high content of alloying elements. These commercial alloy powders were chosen to create a set of dissimilar titanium alloy combinations, to understand the machinability of distinctly different diffusion bonds parings.

The distribution of the dissimilar powder inside the graphite tooling required a custom 3D printed polymer divider to partition the volume inside the graphite mould. The divider geometry can be engineered based on the required materials, properties, and loads and stresses calculated during the design stage. After this, each region of the divider is easily filled with different titanium alloy powders before the divider is then vertically lifted to leave the powders with the corresponding arrangement. This means that the divider is customisable and reusable. 

The 3D printer structure used in this work was designed to divide the inner cylindrical mould volume in eight equivalent arc sections. Figure 1a shows the powder layout after extracting the divider and the layout location of the five titanium alloys in the eight available sections. Two circular sector regions of Ti-64 were designed into the *Mul*Ti-FAST billet in opposite halves to (1) act as reference regions for the force feedback data to help map the exact location of the other alloys and (2) ensure there was no drift in force response circumferentially around the *Mul*Ti-FAST billet. As all the alloy pairings and diffusion bonds are sintered in the same solid billet under the same processing conditions, all the results are easily comparable, as the processing parameters are identical. This is a similar approach as the one used by previous researchers using the technology FAST to join dissimilar powders [18,19].

The feedstock used in this study were oversize powders from the manufacturing processes Electrode Induction Gas Atomisation (EIGA) and Plasma Rotating Electrode Process (PREP). Such powder was deemed unusable by the additive manufacturing community. A Mastersizer 3000 laser diffraction particle size analyser (Malvern Panalytical LTD, Malvern, UK) was used to determine the powder size distribution (PSD). The results from this analysis are summarised in Table 1

Elemental analysis of the powder composition was also performed to make sure the composition of the powder selected was within the specified limits in the standards. The weight (%) composition of the powders used in this study are listed in Table 2.

The Ti-64 and Beta C powders used met the standards for Grade 5 and Grade 19 specifications for titanium and titanium alloy bars and billets (ASTM B348/B348M—19, 2015 [44]). The Ti-6242 powder comply with AMS 4919J standards [45] and Ti-5553 had a higher content of oxygen when compared with Boeing Material Specifications [46]. 

### 2.2. Processing Conditions

The *Mul*Ti-FAST billets produced and analysed in this study were produced with the same processing conditions. The heating rate used was 100 °C /min up to 900 °C and then lowered to 50 °C/min until the dwell temperature was reached to reduce overshoot caused by the thermal inertia. While the sample was heating, the pressure increased from 1 MPa to 25 MPa. The dwell temperature was 970 °C for 25 min and the samples were cooled down inside the vessel at a rate of ~35 °C /min. All the processing was performed under vacuum conditions to avoid oxidation of the powder.

### 2.3. Extraction of the Samples and Characterisation

The characterisation of the machined surface and subsurface damage was carried out using a scanning electron microscope (SEM) FEI Inspect F50 microscope, manufactured by FEI company, Hillsboro, USA. The backscattered electron micrographs were captured at an accelerating voltage of 20 kV with a spot size of 4.0. Figure 1d depicts the location of where samples for microstructural analysis were extracted from the *Mul*Ti-FAST billet. Samples were extracted 15 mm from the outer edge from an 80 mm diameter disc by precision cutting sectioning. Furthermore, the bond was placed in the middle of the sample, perpendicular to the radial plane. 

The hardness evolution at the bond regions was analysed through line hardness profiles diagonally to the bond location. The measurement line length was set to 1000 μm, with the bond in the centre. This was performed in order to capture the hardness profile at the bond region as well as the bulk materials. The hardness measurements (HV 1 kgf) were performed on a DuraScan 70 G5 Microhardness Indenter (Struers, Salzburg, Austria).

### 2.4. Machining and Force Analysis

The face-turning machining operation was selected to measure the cutting forces across dissimilar titanium alloy diffusion bonds in the *Mul*T*i*-FAST billets. Turning was used (as opposed to milling, for example) for the following reasons: (1) the aspect ratio of the *Mul*T*i*-FAST billets means that the faces of the manufactured discs present a larger area to be machined than the outer wall of the cylinder; (2) clamping the cylindrical sample through the outer wall presents a more stable machining and rotational configuration; (3) the tool path is always perpendicular to the radial bonds; (4) the diffusion bond regions are crossed several times during the complete face turning operation enabling consistent bond effects in the machining forces to be studied effectively. A set of soft jaws was specifically manufactured to ensure a stable system during machining.

**Figure 2:**
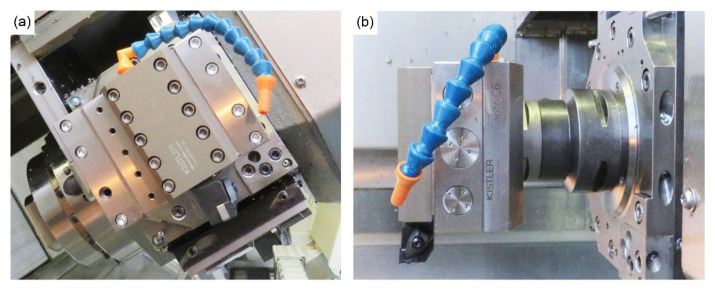
Photographs of the (**a**) front and (**b**) side of the Kistler 9129AA dynamometer plate installed in the WFL M100 MillTurn machining centre with the tool holder and insert in place prior to the machining operation.

The machining trials were conducted on a WFL M100 MillTurn CNC machining centre (WFL Millturn Technologies, Linz, Austria). The machining forces were measured using a dynamometer supporting the tool holder and connected to the machine arm. The dynamometer plate used for this study is a Kistler 9129AA (Kistler, Winterthur, Switzerland) containing eight piezoelectric sensors that measure the forces in the three spatial axes. This is a direct measurement of the machining forces, with a direct correlation of the forces exerted into the tool with the voltage signal provided by the piezoelectric sensors. These signals are collected through the multi-channel charge amplifier (Kistler Type 5070, Kistler, Winterthur, Switzerland) and the Data Acquisition system (Kistler DAQ Type 5697A1, Kistler, Winterthur, Switzerland) and saved via Kistler’s Dynoware software V3.1.0.0. (Dynoware, Winterthur, Switzerland). The dynamometer installed in the CNC machining centre with the tool holder and insert before connecting the umbilical cable is shown in Figure 2. The tool inserts selected for this study were CNMG 12 04 08 SM 1115 manufactured by Sandvik (Stockholm, Sweden), which have a tool radius of 0.8 mm.

The face-turning operation was conducted at a constant RPM (G97) rather than a standard constant cutting speed operation (G96). This was for two reasons: (1) If a constant surface speed is considered due to the relatively small diameter of the sample, the maximum saturation RPM of the centre will be achieved, and hence the G96 constant surface G-code command will be ineffective once this saturation RPM is achieved. (2) The bonds are crossed several times during the face turning operation. This means that the influence of the bond on the machining forces can be analysed at different cutting speeds. The speed decreases at radial locations close to the centre. 

The acquisition rate used for collecting the data was set to 30 kHz, and the machining parameters selected for this test were 0.15 mm of depth-of-cut (*ap*), feed of 0.15 mm/rev (f_rev_) and a constant RPM value of 137. This means that the angular resolution of the test is a constant 36.5 points per degree. In terms of linear resolution (points per unit of length travelled by the insert), it increases towards the centre. The smallest linear resolution in the outer locations of the turned face is 52.3 points per mm. The resolution increases towards the centre at a rate of $40/R_{i}$, where $R_{i}$ is the radius from the tool to the centre of the billet at a specific time during the machining operation.

### 2.5. Analysis of Machining Force Feedback Data

**Figure 3:**
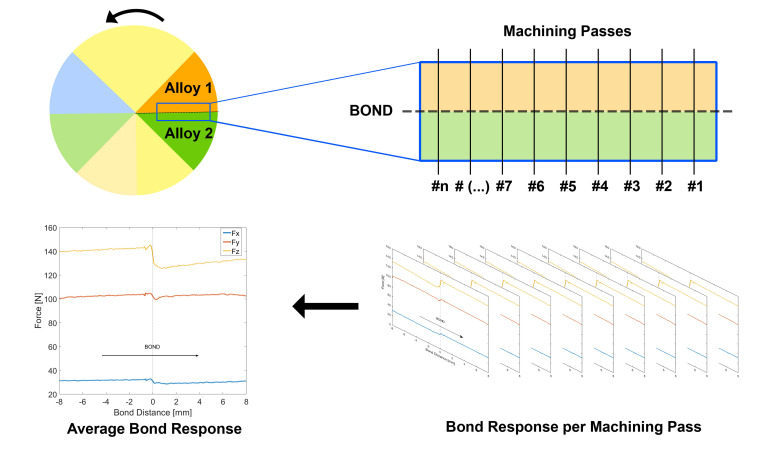
Graphical representation showing where data is extracted from and how the average forces in the bond are calculated.

Due to the nature of the face-turning operation and the orientation of the diffusion bonds, each bond is traversed by the tool once per rotation. This means that it is possible to calculate the average response of the machining forces (per rotation) to evaluate the behaviour of the machining forces across the bond. These values were calculated from a sub-dataset comprising a geometric region 16 mm wide, with the bond in the centre. The individual bond force response in the three axis was then calculated as a function of the bond distance per machining pass across the bond and then averaged. This ensures that the machining effects, when the tool insert crosses the bond, displayed in such plots are consistent and unaffected by individual events, such as vibrations and chatter. A graphical representation of how these datasets were extracted is shown in Figure 3.

## 3. Results

In order to understand the machining challenges of manufacturing multi-material billets made with dissimilar titanium alloys, the microstructure of each alloy and bond region was fully analysed. This was complemented by hardness profiles measurement across the diffusion bond regions and the analysis of the average force response measured during machining at the bond locations. 3D optical surface topography metrology analysis and secondary electron, high resolution topography reconstruction is also presented in this section.

### 3.1. Diffusion Bond Characterisation

#### 3.1.1. Microstructure Analysis

**Figure 4:**
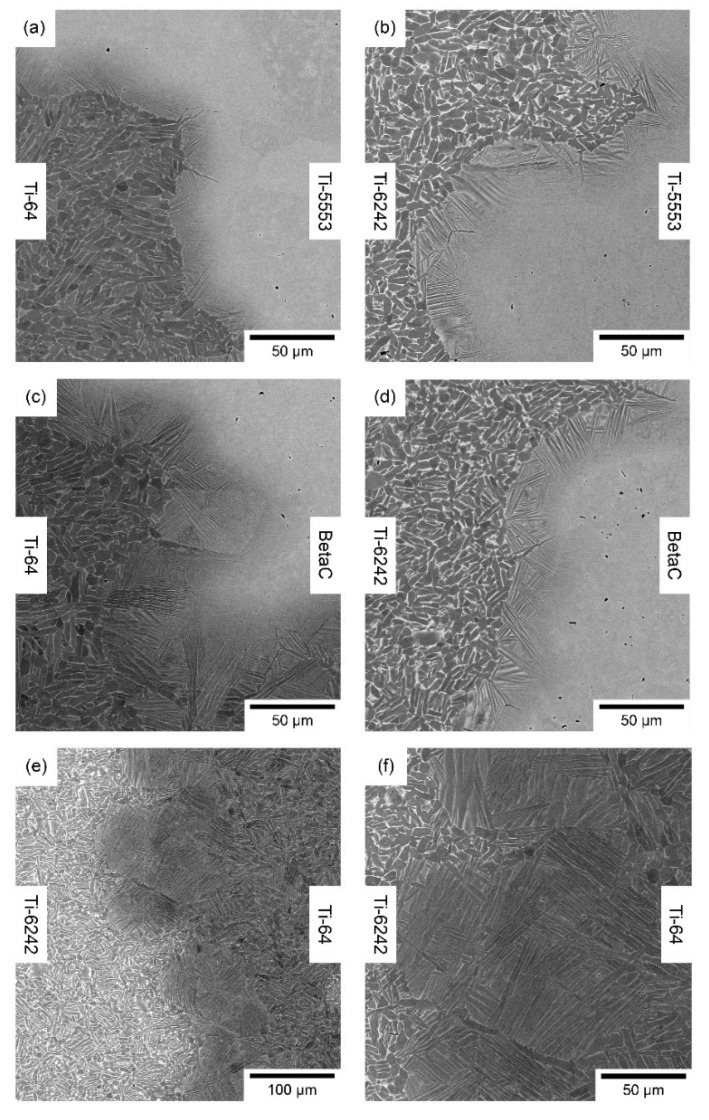
Backscatter electron micrographs of a range of titanium alloy diffusion bond pairings from the *Mul*Ti-FAST billet, processed at 970 °C and for a dwell time of 25 min. (**a**) Ti-64/Ti-5553, (**b**) Ti-6242/Ti-5553, (**c**) Ti-64/Beta C, (**d**) Ti-6242/Beta C, (**e**) Ti-6242/Ti-64 at low magnification and (**f**) Ti-6242/Ti-64 at high magnification.

The backscatter electron (BSE) images of the microstructure (Figure 4) show the microstructural development for different titanium alloy diffusion bond pairings from *Mul*Ti-FAST billet. FAST processing these alloys in the two phase α + β region (i.e., subtransus processing) leads to Ti-64 and Ti-6242 developing a combination of equiaxed primary α and a lath-like structure in the β transformed regions. In contrast, Beta C and Ti-5553 were FAST processed above their respective β transus temperatures, in the single phase β region, and as a result the microstructure is a standard fully transformed large-grained structure. 

An interesting microstructural development occurs at the diffusion bonded alloy interface when an α or near α is bonded to a β or near β titanium alloy: a finer acicular α structure is developed perpendicular to the bond in the β region of the interface, reported previously by Pope et al. [18]. The microstructures generated at the bond between an α and a β titanium alloy was also studied by Motyka et al. [47].

In the Ti-64/Ti-6242 bond interface, a distinct change in microstructure is observed with a supertransus-like microstructure that can be influenced by the chemical composition of the alloy additions diffusing through the chemical graded bond. As a consequence, the local chemistry has a β transition temperature lower than Ti-64 and Ti-6242 and lower than the FAST processing temperature [19].

#### 3.1.2. Hardness Profile Evolution

**Figure 5:**
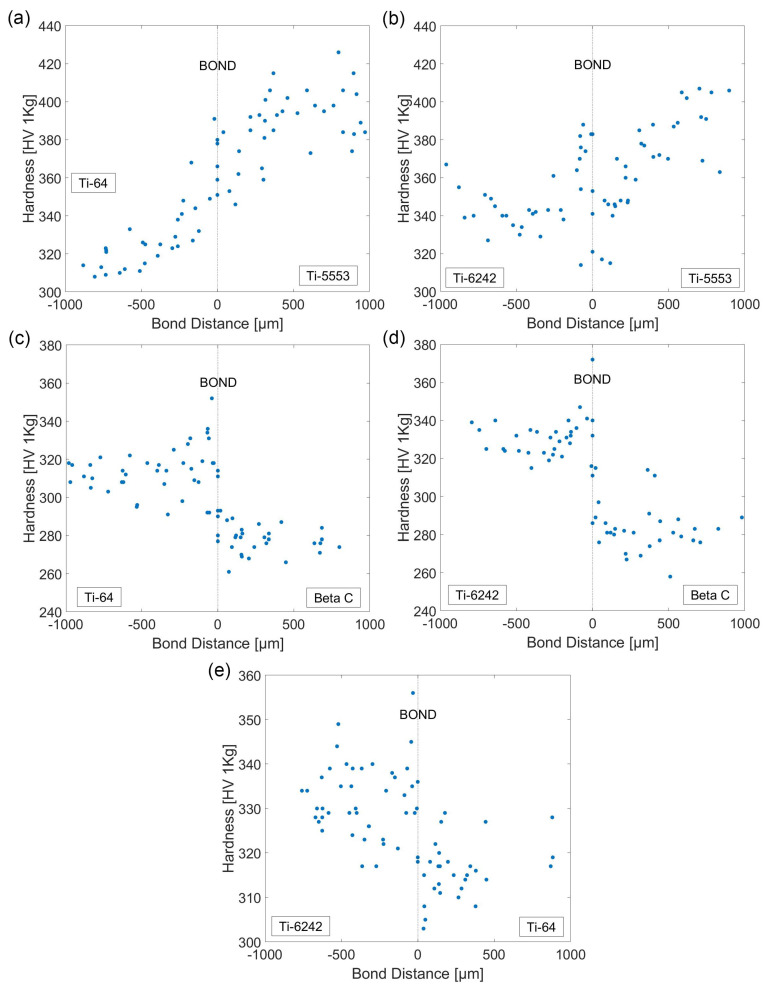
Hardness profiles (HV 1 kgf) for a range of titanium alloy diffusion bond pairings FAST processed at 970 °C and for a dwell time of 25 min. (**a**) Ti-64/Ti-5553, (**b**) Ti-6242/Ti5553, (**c**) Ti-64/Beta C, (**d**) Ti-6242/Beta C and (**e**) Ti-6242/Ti-64.

The gradual variation in the hardness profile across each diffusion bond pairing is shown in Figure 5. From these results, the hardness profiles can be classified into three different categories: (i) maximum hardness found at the interface, (ii) steep change at the bond and, (iii) no appreciable change between both materials.

In the two bond pairings where Ti-5553 is present (i.e., Ti-64/Ti-5553 and Ti-6242/Ti-5553), the level of hardness values across the bond is smoother compared to the other alloy pairings, with no abrupt changes in the measured hardness values. The hardness levels in the bond transition region are within the limits defined by the bulk hardness of both materials. The high strength Ti-5553 also presents the highest hardness values of all the alloys analysed, with a maximum hardness value of ~420 HV.

The bonds that contain Beta C show a different trend: the maximum measured hardness was found at the bond interface region. The Ti-64/Beta C and the Ti-6242/Beta C bond pairings exhibited a hardness peak value of ~360 HV and ~370 HV, respectively. These two hardness profiles show a more abrupt change in hardness, with the lowest hardness values being in the Beta C bulk region.

Finally, the Ti-64/Ti-6242 bond represents the alloy combination with the most similar machining response and microstructural features. In this case, the largest hardness value measured was also found near the bond region; however, due to the data scatter, specifically in the Ti-6242 regions, this peak is not as prominent as observed in other alloy bond pairings.

### 3.2. Bond Average Force Response

**Figure 6:**
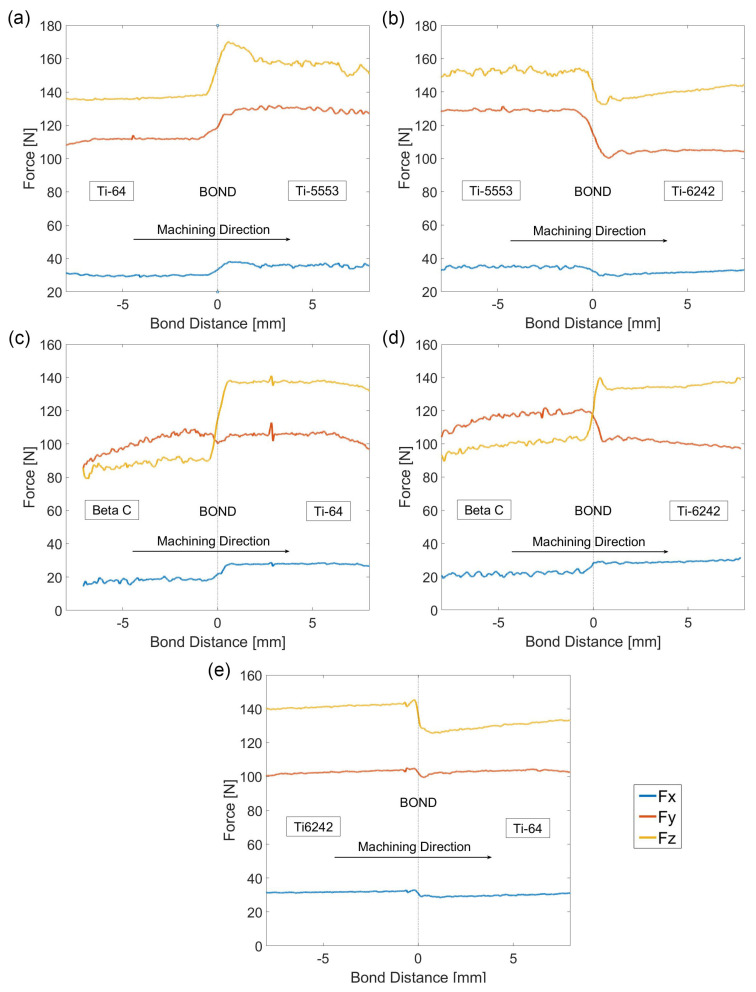
Plots presenting the average machining forces across the diffusion bonds response for Fx, Fy and Fz. (**a**) Ti-64/Ti-5553, (**b**) Ti-6242/Ti5553, (**c**) Ti-64/Beta C, (**d**) Ti-6242/Beta C and (**e**) Ti-6242/Ti-64.

The machining forces across the titanium alloy diffusion bond pairings are shown in Figure 6. The results presented in Figure 6 show that in all the studied alloys, the maximum force value was measured in the *z*-axis, longitudinal force (i.e., force parallel to the rotational axis), followed by the *y*-axis, machining force) and finally the *x*-axis, feed force (force parallel to the feed axis). However, in the case of alloy Beta C, the average Fy force is always greater than Fz. As this effect occurs even at bulk alloy regions, away from the bond, this effect is attributed to the machining response of Beta C rather than a bond related effect.

In terms of force disturbances at the bond, the combination of alloys that presented the best compatibility was Ti-64/Ti-6242, as the maximum change in force presented in Fz was only ~7%. This is a consequence that both alloys are very similar in chemical composition, and hence they have similar β transus temperatures. Therefore, the chemical gradient and the microstructural development between both alloys processed under the same FAST parameters is relatively similar; hence, both alloys react similarly during machining. The largest fluctuation in cutting performance was found in the Beta C/Ti-64 diffusion bond pairing, with a force deviation of up to 40% registered in Fz.

However, in several of the diffusion bond pairings, the change in cutting performance was not smooth: sometimes, a consistent increase (or decrease) in the machining forces was found when the tool traversed across the bond. In the case of the Beta C/Ti-6242 diffusion bond, consistent increases in force were measured immediately after the tool traversed the bond. This similar behaviour was also observed in the Ti-5553/Ti-64 diffusion bond pairing. The opposite was also observed in the Ti-5553/Ti-6242 bond, where a consistent decrease in the machining forces is measured as the tool engages with the Ti-6242 region. Such an effect is more pronounced in the Fz and Fy signals.

An analysis of the force variability in the bulk regions far from the bond was also performed. The standard deviation of the forces measured by the dynamometer in the three spatial axes are listed in Table 3.

The standard deviation analysis shows that the alloy that presented more constant cutting forces and less variability is Ti-64, with a maximum standard deviation of 2.31 measured in the *z* axis. A similar result is shown in Ti-6242, specifically in the *z* axis. However, for the metastable β alloys, FAST processed in the single β phase, a larger standard deviation value is presented which can be related to the differences in the microstructural development that occurs over the β transus temperature. This effect was reported by Suárez Fernández et al. [48] for β processed titanium alloys.

### 3.3. Directionality Effects on Bond Machining Response

**Figure 7:**
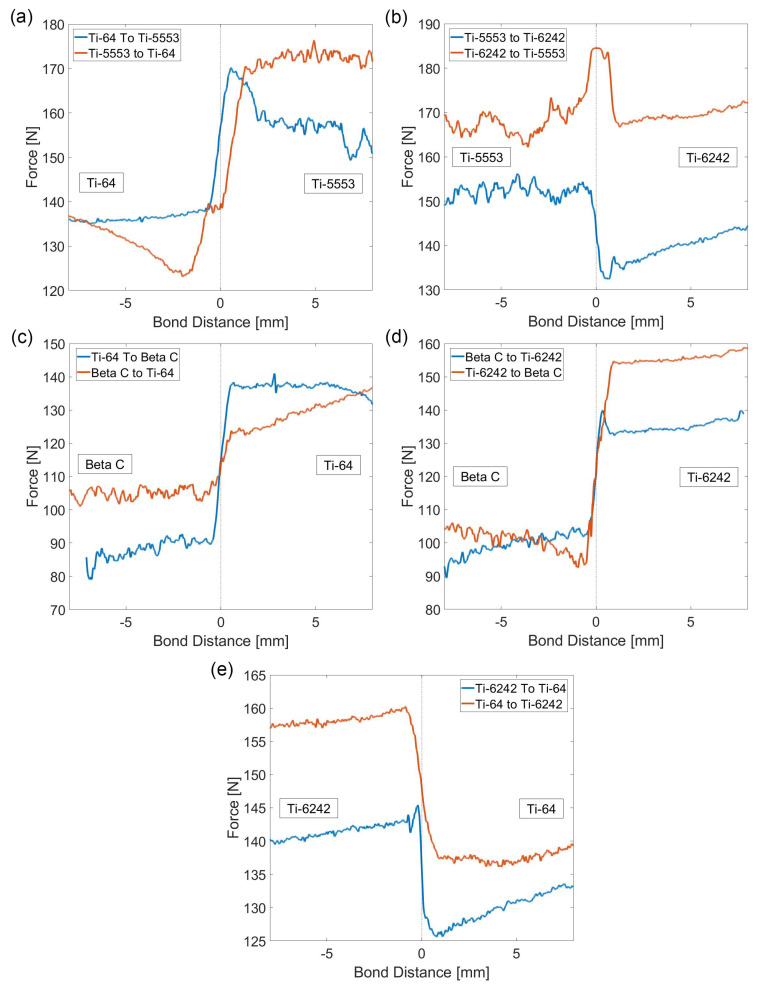
Plots of the average Fz profile for both machining directions for each titanium alloy bond pairing. The dash line at 0 represents the location of the bond. (**a**) Ti-64/Ti-5553, (**b**) Ti-6242/Ti5553, (**c**) Ti-64/Beta C, (**d**) Ti-6242/Beta C and (**e**) Ti-6242/Ti-64.

In order to further investigate the effects of the bond and alloy pairings on the machining forces, a directionality study was carried out. This is required to understand the best machining strategies of multi-material or functionally graded titanium parts. An efficient and equivalent approach to machining across the diffusion bonds in both directions was achieved by machining both sides of the *Mul*Ti-FAST billet. During the investigation, the machining parameters remained constant and there was no need to invert the tool shank or modify the machining G-code. Figure 7 shows the evolution of the Fz force during the machining operation across all five diffusion bond pairings with respect to machining direction. The parent material behaviour and force fluctuation on both sides of the diffusion bond were unaffected by machining direction. However, the bond region between the alloys was certainly sensitive to the cutting direction. 

In the case of the Ti-5553/Ti-64 bond pairing, when the tool traversed from Ti-64 into the Ti-5553, a sharp increase in force was observed immediately after crossing the bond in the Ti-5553 region. This peak force was an absolute maximum, registering a value of ~170 N, greater than the average bulk Fz value for both alloys. However, when the direction was reversed (i.e., from Ti-5553 to Ti-64), a sudden drop in force was registered in the Ti-64 region immediately after traversing the bond. 

Similar behaviour was observed in the Ti-5553/Ti-6242 bond pairing: an increase in the force and a force plateau appears at the interface region when the tool travels from Ti-6242 into the Ti-5553. However, when the machining direction is reversed, a sudden drop in force appears in the Ti-6242 region, after crossing the bond.

The Beta C/Ti-64 bond pairing shows more consistent reversible behaviour, where the machining direction is not as important with respect to the bond region, in terms of forces. A constant transition between the bulk machining response is presented, regardless of the machining direction, with a consistently larger Fz value measured in the Ti-64 region. However, the Beta C/Ti-6242 bond pairing does exhibit machining direction effects. When the tool crosses the bond from the Ti-6242 region into Beta C, the lowest Fz value is found immediately after the interface, which is below the bulk Beta C average Fz value. On the contrary, when the machining direction is reversed, a noticeable peak in the force appears in the Ti-6242 side.

Finally, the Ti-6242/Ti-64 bond pairing only exhibits a small peak when the tool travels from Ti-6242 into Ti-64. This peak appears just before the sharp decrease in forces in the Ti-64 region. In the reversed case, no abrupt changes in force are observed, apart from the inherent change in machining behaviour between the two alloys.

### 3.4. Surface Topography Maps and Digital Fingerprint Reconstruction of Microstructures

In order to evaluate if the effects found in the directionality analysis in Section 3.3 are also evidenced in the surface topography features, a 3D topography map of the as machined surface was constructed. To generate this data, a variable high resolution optical microscope with focus variation for dimensional metrology (Alicona Infinite Focus SL, Alicona Imaging, Raaba, Austria) was used. A topographic map was constructed for each analysed diffusion bond pairing and per machining direction.

In addition, the fingerprint reconstruction of the bonds was also performed for the identical locations. These fingerprints were reconstructed by synchronising the machining forces with the tool path parametrisation developed by Suárez Fernández et al. [49]. 

**Figure 8:**
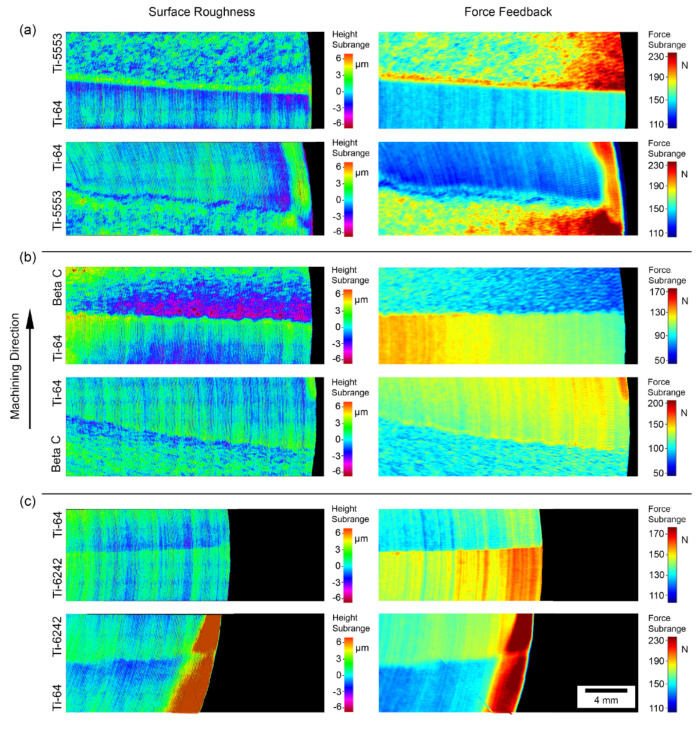
Surface topography and force feedback reconstruction plots at equivalent locations comparing the force and the surface roughness of the Ti-64 diffusion bond pairings in both machining directions. (**a**) Ti-64/Ti-5553, (**b**) Ti-64/Beta C and (**c**) Ti-64/Ti-6242.

Figure 8 shows the topography surface and the force feedback reconstructed diagrams from the bonds containing the alloy Ti-64 in both machining directions. The results observed in this case for the bond with Ti-64 can be compared to the bonds containing Ti-6242. 

An interesting result is that the topographic maps and the reconstructed digital fingerprint diagrams for the machining forces are relatable. The bonds and features are easily identifiable in all the analysed alloy bond pairings and directions. However, it is important to highlight that although the maps are similar, the reconstructed fingerprint carries information about the machining dynamics, while the topography map is created statically using optical methods. Therefore, producing equivalent resolution and perfectly synchronised maps, as shown in Figure 8, is challenging.

From the maps shown in Figure 8, it is clear that most of the bonds are aligned with the radial direction. The alloy bond pairing that deviated the most from the radial direction is Ti-64/Ti-5553. This deviation from the radial direction is related to the differences in original powder size and compressibility ratios, as well as any misalignments when extracting the powder divider prior to FAST processing stage. The face-turning operation was conducted at a constant RPM value and therefore the cutting speed increased slightly towards the centre. It is interesting to note that during machining, the variation of the cutting forces was not large, suggesting that the cutting stability and dynamics were constant throughout the machining trials. 

In terms of resolution and features, the Alicona scan presents a constant higher resolution per unit of area, compared to the reconstructed fingerprint map of the machining forces. In the latter case, the resolution increases due to the reduction in cutting speed towards the centre, as the acquisition data rate is kept constant throughout the trials. Moreover, regarding the feature resolution, the colour scheme used for the topographical maps is more suitable for both materials, as the different features at each side of the diffusion bond are at a similar height. On the contrary, the colour scheme used for the reconstruction of the machinability fingerprint maps is more difficult to apply in order to reveal all the features, as the force ranges on both sides of the bond can vary, and hence a compromise has to be made.

These force maps show the same trends presented on the directionality analysis above (Figure 7). For example, the first bond pairing of Ti-5553/Ti-64 presents a consistent increase in forces in the Ti-5553 region, immediately after the tool traverses the bond, when machining from the Ti-64 side. In the same region where the forces increase, the topographic map also shows an abrupt change in surface height (coloured in green parallel to the bond). On the contrary, an inverse effect is shown when the direction is reversed, where a decrease in the forces and a consistent reduction in surface height immediately after the tool crosses the bond.

The comparison between the fingerprint and topographical maps also shows that most of the interesting features occur immediately adjacent to the bond, because the regions consisting of only one single alloy show identical steady-state cutting behaviour and surface topography, regardless of the machining direction. These maps also show a correlation between the fingerprint reconstruction carried out with the machining forces and the surface maps.

### 3.5. Surface Analysis Damage Assessment

**Figure 9:**
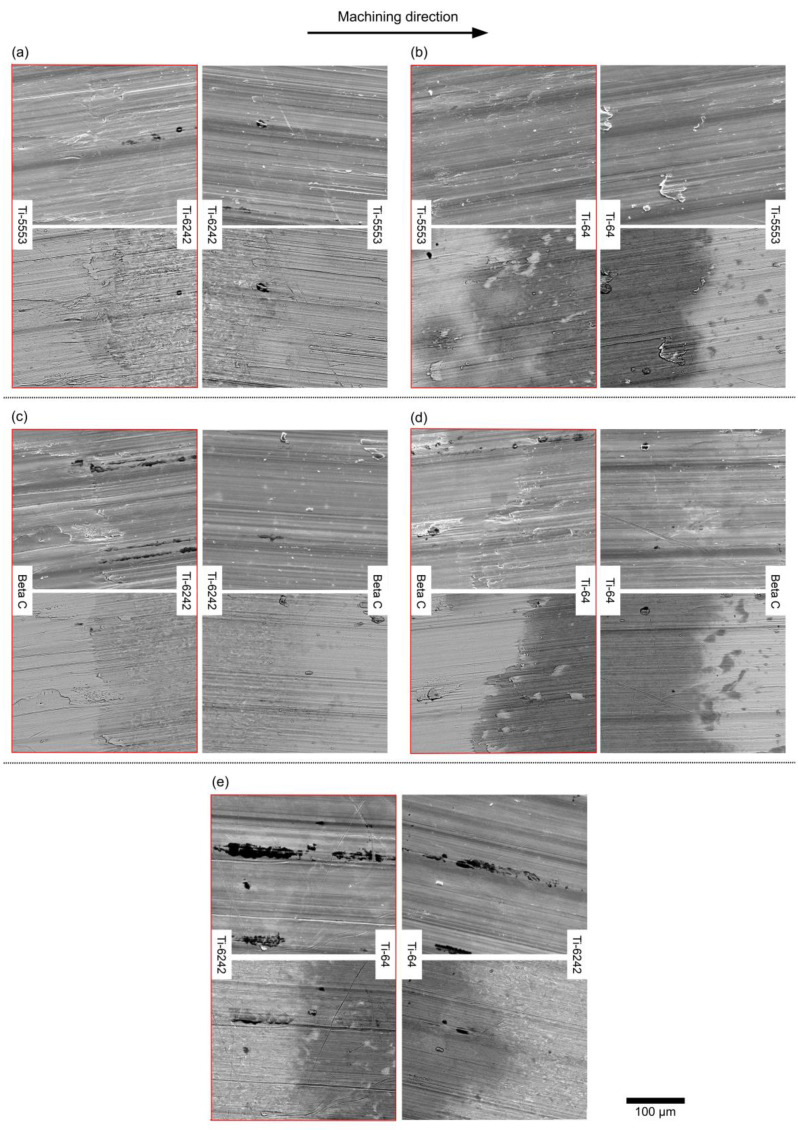
Scanning and backscattered electron micrographs of the diffusion bond pairings showing the machined surface of the bonds. For each alloy pair, the secondary electron micrographs are on the top row and the backscatter electron micrographs are on the bottom row. (**a**) Ti-6242/Ti-5553, (**b**) Ti-64/Ti5553, (**c**) Ti-6242/Beta C, (**d**) Ti-64/Beta C and (**e**) Ti-6242/Ti-64.

The machined surface generated in the five alloy bond pairings from the machining trials is shown in Figure 9. Secondary and backscattered electron images are presented for each bond and machining direction. The compositional Z contrast from the backscattered electron images enable the exact position of the bond to be located, so the damage can be accurately correlated to the bond position.

Overall, there is always damage when machining from the metastable β to the α + β alloys (micrographs inside the red box). This damage is shown clearly in Figure 9a–d, where there is a combination of pick-up (which is re-deposited built-up edge material from tool cutting edge) in the exact location of the bond as well as a distinctive change in surface topography (valley formation). However, this damage is not observed in Figure 9e where the bond is composed of two similar alloys. When machining from the α + β to the metastable β alloys, there is no specific damage observed in any of the bond pairings.

The damage produced in the bond is not linked to a specific microstructure. The results shown in Figure 9 have also been observed in other samples with bonds produced from the titanium alloys—Beta C and Ti-6242—processed at 1100 °C for 60 min and 25 min. This temperature is above the β transus temperature of both alloys, and thus, the microstructure of Ti-6242 is fully lamellar instead of the equiaxial microstructure presented in this study. Furthermore, the Beta C grains will be larger than the microstructure presented previously in Figure 4, due to the enhanced grain growth promoted by the increase in FAST processing temperature. The longer processing time, from 25 to 60 min, further enhances grain growth in both microstructures.

### 3.6. Subsurface Microstructural Damage at the Diffusion Bond Pairings

The cross section of the diffusion bonds was analysed for any potential machining-induced subsurface microstructural damage. Results show that the type and intensity of subsurface damage, manifested as swept grains and alpha laths, is within the expected level for titanium alloys when being machined under such conditions [50]. Under the selected turning parameters, the introduction of a multi-material bond does not increase the amount of subsurface damage when directly compared to the bulk material. Furthermore, the machining direction is another parameter that does not appear to influence the subsurface damage levels. 

**Figure 10:**
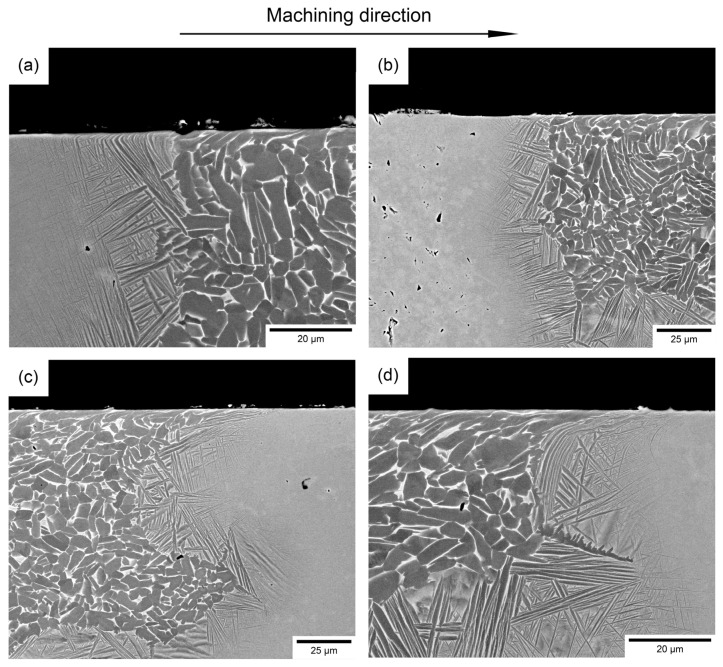
Backscattered electron micrographs of the cross section-induced damage for alloy bond pairings consisting of (**a**,**c**) Ti-5553/Ti-6242 and (**b**,**d**) Beta C/Ti-6242.

Figure 10 shows four bonds: the interface between Ti-5553 and Ti-6242 bond pairing (Figure 10a,b) and interface between Beta C and Ti-6242 bond pairing (Figure 10c,d). In both bonds, swept grains are observed down to 5–10 μm into the surface and there is minimal change in the swept grain characteristics with respect to the machining direction.

## 4. Discussion

The focus of this work is to a gain valuable insight into titanium functionally graded and multi-material machining. Analysis of the forces gathered during the operation, surface defects and subsurface microstructural damage provide important information for the design of smart machining strategies of multi-material components. In this section, we aim to discuss the severe plastic deformation mechanisms and force response that explain the variation in behaviour for the different alloy pairings.

### 4.1. Surface Finish Defects at the Bond

Table 4 summarises the results presented in the results section and the following information about the bonds: if a local maximum in the forces was recorded at the bond; if there is a formation of a valley; if damage on the surface was present.

From the results presented in the previous section, no clear relationship exists between the maximum local forces measured at the bond region in the *z* axis and the machining and surface damage found in the sample, which is summarised in Table 4. However, in the case of the bond pairings that incorporate Ti-5553, the surface topography maps correlate well with the damage features found in the bond region. This correlation was not observed in bonds containing the metastable β alloy—Beta C.

The main variable that is found to correlate well with the surface machining-induced damage is the machine direction. Consistent features were found in the bond region when machining from a metastable β alloy through to an α + β alloy.

Although the microstructure resultant of the FAST processing conditions influences the amount of pick-up generated and the severity of the damage, it does not change the overall damage characteristics in the bond when machining from a β to an α + β alloy. This has been observed in two supertransus bonds with different dwell times in none published research.

Notice that when joining similar titanium alloys such as the Ti-64/Ti-6242 bond pairing, no noticeable damage was found regardless of the machining direction.

**Figure 11:**
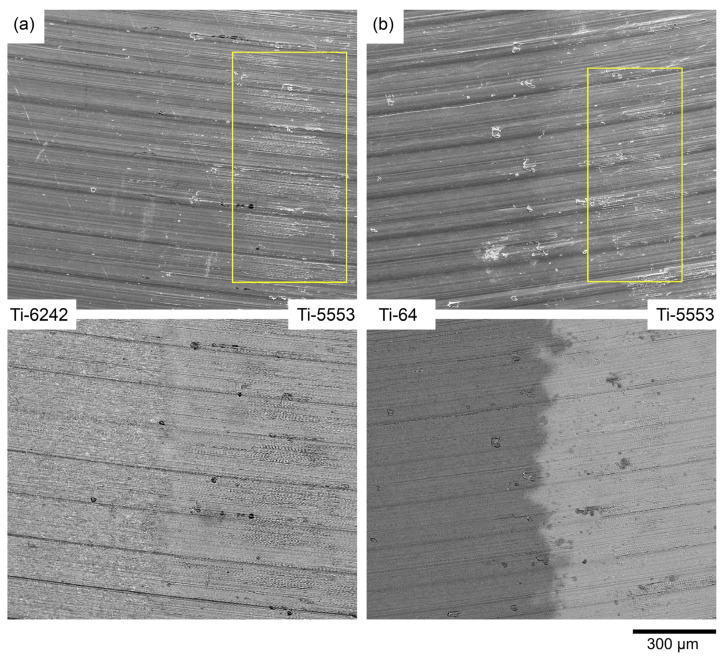
Secondary electrons and backscattered electron micrographs for the pick-up of redeposited built-up edge material from the cutting edge at the diffusion bond region for the pairings of (**a**) Ti-5553/Ti-6242 and (**b**) Ti-5553/Ti-64.

The surface finish, when machining from Ti-5553 to Ti-64, presents clear pick-up features at the bond region (Figure 9). However, when machining from Ti-64 to Ti-5553, there is no evidence of surface damage adjacent to the bond, apart from pick-up located randomly on the surface. This result does not correlate well with Figure 7 and Figure 8 where a peak at the surface and a peak of force was observed at the bond region. The challenge with the topographical map and the force feedback data is that it is difficult to pinpoint the exact location of the bond. From Figure 11, the increase in the surface finish does not occur in the bond: it occurs at approximately 200–300 μm from the bond line, as depicted by the yellow boxes. This distance is greater than the expected diffusion distance from the bond for a subtransus microstructure [19]. The Z-contrast brightness of the pick-up in the backscattered electron micrographs in Figure 11 shows that it is not exclusively from the Ti-64, as it consists of traces from the metastable β alloy also. It is a possibility that changing alloy chemistry, and hence changing the reaction between the tool and workpiece material (and propensity for built-up edge (BUE)), results in an abrupt “cleaning” of the tool tip through the scraping of the BUE from the cutting edge onto the machined surface, possibly reducing the integrity of the component in such locations [43].

### 4.2. Dynamic Effects and Machining Response

There has been previous research comparing the machinability between a metastable β alloy and α + β alloy. The machining of Ti-5553 tends to create higher forces compared to Ti-64 [51,52,53]. This is caused because the thermal conductivity of Ti-5553 (22 W/mK) is 30% higher than that of Ti-64 (15 W/mK) at 700 °C [53]. Lower thermal conductivities promote the formation of chip segmentation, which reduces the reaction force. However, a higher thermal conductivity transfers the heat from the material to the chip more rapidly, which reduces the degree of softening occurring in the material during machining [53]. In addition to this, several studies have shown that the high strength, metastable β alloys, such as Ti-5553 and Beta C, tend to adhere more to the cutting face of the tool compared to Ti-64, which creates larger BUE chip thicknesses [53,54,55]. This correlates well with Figure 9, where there is a large amount of pick-up at the bond when machining from a metastable β to Ti-64. Furthermore, Rashid et al. [56] observed the formation of built-up edge (BUE) in β alloys is higher for more ductile workpieces.

**Figure 12:**
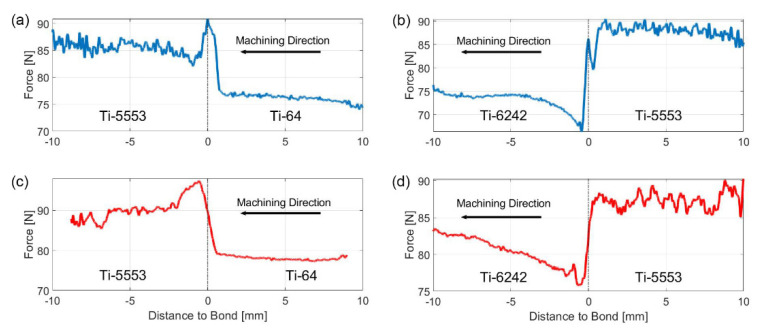
Plot of the forces generated in the machining operation of a FAST diffusion bonded sample made of Ti-5553 with Ti-64 and Ti-6242. (**a**,**b**) machined with a WFL M100 Millturn, (**c**,**d**) machined with a MAG Hawk 300 Lathe.

From all this information, it is clear that the effects observed when machining multi-materials are influenced by the workpiece microstructure and/or the CNC machine. Some of the *Mul*Ti-FAST billets were machined on two different CNC machines, to check that the trends in the forces were consistent in different equipment. As shown in Figure 12, the same force trends were observed for all the bonds tested, this clarifies that the directionality effects are not linked to the CNC machine used for most of the tests.

In previous investigations, it has been observed that there are multiple factors affecting the machined surface quality of titanium alloy components in machining operations, such as tool shape, geometry and tool wear, temperature, tool coating, feed rate, cutting speed, depth-of-cut and BUE formation [57]. Some of the parameters such as tool geometry, tool coating and tool wear can be discarded as an influencing factor, because the two alloys were machined with the same tool during the same machining operation. The rest of the parameters cannot be discarded because there is not enough data or the data is difficult to measure while processing. For example, the feed rate, cutting speed and depth of cut should be the same for both alloys, but the machinability of α + β alloy and metastable β alloys is different. Hence, it is possible that the Numerical Control (NC) of the equipment used, had to adjust the settings in real-time to maintain the same selected cutting parameters. If the tool decreases the speed during the initial stages of metastable β alloy machining, it could take a fraction of a second for the machine to increase the power to maintain the same speed. In that fraction of second, as the machine is adjusting its settings, the speed decreases and both the pick-up and reaction forces increase. All this relates well with the observations from the Alicona and force feedback maps, as well as the pickup generated in Figure 8. However, this will not be possible to demonstrate without being able to obtain the data of the actual speed of the machine. 

## 5. Conclusions

This research provides valuable insight for the manufacturing of future multi-material components. This was achieved by analysing the compatibility of different titanium alloy pairings, processed under identical conditions and investigating the relationship between the manufacturing variables and the development of undesired features that could have detrimental effects in the final component’s performance.

Regarding the diffusion bonded pairings, no microstructural or joining defect was found in the bond interface. This result is in line with the previous published results found in the literature. For the manufacturing process investigated in this work, results are directly related to the pairing compatibility and machining parameters. This is supported further by the repeatability of the results seen in other CNC machining centres. 

This work presents for the first time an integral analysis linking force signals, surface topography and surface and subsurface damage features of a bonded material. 

The results and conclusions highlight the potential issues found when machining multi-material components and will serve as a basis for future studies in the subject. The conclusions obtained from this work are the following:

- Field-assisted sintering technology can be used to successfully join more than five alloys in the same billet without noticeable defects in the bond.
- The machining direction influences the forces generated in the bond, meaning that opposite direction force profiles are different.
- A direct relationship has been found between the surface roughness and the forces in the bond when machining bonds containing Ti-5553. This is not the case for the bonds with Beta C in it.
- The machining direction can have a direct impact on the surface damage found. Lower damage levels are reported when machining from an α + β alloy to a β alloy compared with the opposite case. This is consistent for all the bonds consisting of α + β and β alloys regardless of the microstructural development in both alloys. This means that this effect could be linked to chemistry compatibility.
- Little difference was reported in the bonds consisting of similar alloy types, such as the pairing of Ti-64 and Ti-6242.
- The directionality force trends presented in this study are consistent for different CNC machines, ruling out the possibility of errors induced by the CNC machining controller.

The results and conclusions highlight the potential issues that could arise from designing and manufacturing complex functionally graded materials and some best practices to maximise part quality and minimise deleterious effects in component performance. This has important implications as the results presented in this work could be extrapolated to other joining techniques.

## Figures and Tables

**Table 1:** Type and average statistical powder size distribution of the materials used in this study.

Powder Alloy	Powder Type	Dx (10) [μm]	Dx (50) [μm]	Dx (90) [μm]
Ti-64	PREP	61.3	86.7	123
Ti-6242	EIGA	25.2	37.4	53.7
Ti-5553	EIGA	20.7	57.2	140
Beta C	EIGA	42.3	124	292

**Table 2:** Chemical composition from the elemental analysis of the powder feedstock used in this study in weight (%).

	Ti	Al	V	Sn	Zr	Mo	Cr	Fe	Si	C	S	O	N	H
Ti-64	Bal	6.0	3.6	-	-	-	-	0.16	-	0.023	0.01	0.181	0.003	0.0032
Ti-6242	Bal	5.6	-	1.8	4.4	1.9	-	0.05	0.09	0.07	0.01	0.148	0.002	0.0021
Ti-5553	Bal	5.0	5.2	-	-	5.1	2.7	0.39	-	0.015	0.002	0.203	0.016	0.0033
Beta C	Bal	3.5	7.9	-	4.3	4.6	5.4	<0.05	-	0.004	0.002	0.092	0.018	0.0022

**Table 3:** Standard deviation on the Fx, Fy and Fz forces measured in the bulk alloy regions in *Multi*-FAST billet.

Standard Deviation	Ti-6242	Ti-64	Ti-5553	Beta C
Fx	0.95	0.73	0.80	1.28
Fy	3.06	1.62	1.09	6.71
Fz	2.35	2.31	2.96	3.91

**Table 4:** Summary of the findings in the multiple diffusion bonds studied in this work after being machined.

Alloy Bond Pairing (and Machining Direction)	Peak Force in Z	Valley in Surface	Damage at the Bond
Ti-5553 → Ti-6242	No	Yes	Yes (low)
Ti-6242 → Ti-5553	Yes	No	No
Ti-64 → Ti-5553	Yes	No	No
Ti-5553 → Ti-64	No	Yes	Yes
Beta C → Ti-6242	No	Yes	Yes (low)
Ti-6242 → Beta C	Yes	Yes	No
Ti-64 → Beta C	No	Yes	No
Beta C → Ti-64	No	Yes	Yes
Ti-6242 → Ti-64	No	No	No
Ti-64 → Ti-6242	No	No	No

## Data Availability

Data sharing is not applicable.
